# Oral Fluid Testing during 10 Years of Rubella Elimination, England and Wales

**DOI:** 10.3201/eid1610.100560

**Published:** 2010-10

**Authors:** Gayatri Manikkavasagan, Antoaneta Bukasa, Kevin E. Brown, Bernard J. Cohen, Mary E. Ramsay

**Affiliations:** Author affiliation: Health Protection Agency Centre for Infections, London, UK

**Keywords:** Rubella, population surveillance, sensitivity, specificity, oral fluid testing, disease elimination, England, Wales, viruses, research

## Abstract

Surveillance of rubella in England and Wales has included immunoglobulin M testing of oral (crevicular) fluid from reported case-patients since 1994. The need for laboratory confirmation to monitor rubella elimination is emphasized by poor sensitivity (51%, 95% confidence interval 48.9%–54.0%) and specificity (55%, 95% confidence interval 53.7%–55.6%) of the clinical case definition. During 1999–2008, oral fluid from 11,709 (84%) of 13,952 reported case-patients was tested; 143 (1.0%) cases were confirmed and 11,566 (99%) were discarded (annual investigation and discard rate of clinically suspected rubella cases was 2,208/100,000 population). Incidence of confirmed rubella increased from 0.50 to 0.77/1 million population when oral fluid testing was included. Oral fluid tests confirmed that cases were more likely to be in older, unvaccinated men. Testing of oral fluid has improved ascertainment of confirmed rubella in children and men and provided additional information for assessing UK progress toward the World Health Organization elimination goal.

In 1970, rubella vaccination was introduced in the United Kingdom for prepubertal girls and nonimmune women of childbearing age to protect them from the risks for rubella during pregnancy. Although this selective vaccination policy effectively reduced the number of cases of congenital rubella syndrome (CRS) and terminations of pregnancy, rubella during pregnancy continued to occur (*1*). In 1988, measles, mumps, and rubella (MMR) vaccine was introduced for universal vaccination at 13–15 months of age with the goal of eliminating circulating rubella.

A considerable decrease in rubella in young children followed, but in 1993, clinically diagnosed and laboratory-confirmed rubella increased; the increase occurred predominantly in older men who had previously not been offered a rubella-containing vaccine (*2*). Therefore, in November 1994, rubella vaccine was included in a school catch-up campaign to prevent a predicted measles epidemic (*3*). Approximately 92% of children 5–16 years of age received combined measles–rubella vaccine. In 1996, to maintain measles control, a second dose of MMR was recommended for children 5 years of age.

For any disease in the elimination phase, accurate surveillance is necessary to identify reservoirs of infection and susceptible groups (*2*). In 2005, the World Health Organization (WHO) European Region adopted a resolution to eliminate indigenous rubella by 2010 (elimination goal of confirmed rubella incidence <1 per 1 million population) (*4*). WHO has developed a clinical case definition for rubella (*5*), but identification of cases based on clinical suspicion alone becomes less reliable as disease incidence decreases. Therefore, for countries trying to eliminate rubella, laboratory confirmation of all suspected cases is recommended (*4*).

Before 1994, surveillance of laboratory-confirmed rubella in England and Wales was based mainly on detection of immunoglobulin (Ig) M against rubella in serum. However, because rubella infection is usually mild, physicians are reluctant to obtain blood samples for serum confirmation, especially from young children. There is also some reluctance to obtain serum from men because the diagnosis is not of major clinical significance. Oral or crevicular fluid is a noninvasively obtained clinical specimen that is likely to be more acceptable, especially for children, and is safe and easy to obtain (*6**–**9*). Transudates from the capillary bed situated beneath the margin between the tooth and gum are obtained by rubbing an absorptive device between the gum and the cheek. These samples, which are distinguishable from saliva samples, contain mucosal cells that enable detection of the rubella virus by PCR. Methods for obtaining, extracting, and storing oral fluid samples are well established (*7**,**10**–**13*). Detection of rubella IgM in oral fluid has been validated and shown to be ≈90% sensitive and 99% specific compared with detection in serum (*2*). Samples are also suitable for genome detection (*14**,**15*). Therefore, since late 1994, the enhanced surveillance program in England and Wales has relied on oral fluid testing to provide laboratory confirmation for clinically diagnosed cases of measles, mumps, and rubella (however, serum testing is still recommended for confirmation of infection during pregnancy).

An additional increase in rubella incidence occurred during 1995–1998. Reports of rubella peaked in 1996 (a total of 9,081 clinically diagnosed cases were reported) (*16*). This situation offered an opportunity to evaluate the sensitivity and specificity of the WHO clinical case definition for rubella. In addition, we describe the added value of oral fluid testing during the subsequent 10 years of rubella elimination (1999–2008).

## Methods

Since 1988, physicians in England and Wales have been required by law to report clinically suspected cases of rubella to the proper officer at the local health authority (usually a public health consultant in a Health Protection Unit [HPU]). Since late 1994, when a report is received, the HPU sends an oral fluid kit to the primary-care physician or patient for confirmatory testing. The kit is then returned by prepaid envelope to the Virus Reference Department at the Health Protection Agency Centre for Infections for analysis. A request form contains vaccination history and, until July 2003, some brief clinical features (presence of a rash, fever, conjunctivitis, cough, and lymphadenopathy [type not specified]). Oral fluid testing was also used to test cases that were not formally reported as part of outbreaks in 3 universities associated with imported virus from Greece in 1999 (*17*). A similar process is used for measles (and mumps) (*18**,**19*). If there is a strong clinical or epidemiologic suspicion of rubella in samples tested for measles and for measles in samples tested for rubella, dual testing is performed.

Oral (crevicular) fluid specimens, obtained by wiping a specially designed sponge swab (oral test kit [Oracol; Malvern Medical Developments, Worcester, UK]) around the gum margins, were tested for rubella-specific IgM initially by using a solid-phase IgM–antibody capture radioimmunoassay (*20*). After 2002, an in-house assay for rubella IgG was introduced (*21*), and after 2003, all samples taken within 1 week after symptom onset that were negative for rubella IgM and rubella IgG were tested by reverse transcription–PCR for rubella virus (*14*). In 2006, the rubella solid-phase IgM–antibody capture radioimmunoassay was replaced by a commercial enzyme immunoassay (*22*).

Results of testing were sent to the reporting physician, and copies were sent to the relevant HPU. Confirmed cases were defined as samples positive for rubella-specific IgM in oral fluid or detection of rubella virus genome by PCR in persons without a history of receipt of rubella vaccine in the previous 6 weeks. These cases were reconciled with confirmed rubella infections (positive for rubella IgM in serum) reported to the Health Protection Agency Centre for Infections, from laboratories in England and Wales, and duplicates are removed. Since 1999, residual samples from cases reported by local laboratories are requested to be sent to the national reference laboratory for confirmation by an alternative IgM assay (with or without avidity testing) (*23*). Cases with negative results for the second IgM test or with high avidity are then excluded from the confirmed total. Further details (including travel and contact history) are requested, and vaccination status is checked for all confirmed case-patients.

Data obtained during January 1995–July 2003 were analyzed to estimate the sensitivity and specificity of a clinical case definition. The WHO definition of a case of rubella is a generalized maculopapular rash and fever and arthralgia/arthritis or cervical, suboccipital, or postauricular lymphadenopathy (*5*). Because information for arthritis was not routinely obtained, the accuracy of a modified case definition based on rash, fever, and lymphadenopathy was calculated against the standard of presence or absence of rubella-specific IgM in an oral fluid sample. The sensitivity, specificity, and positive predictive value were compared by patient age, sex, and year of report.

Data obtained during January 1999–December 2008 from 3 sources (clinically reported cases, confirmation in oral fluid samples, and laboratory reports) were then compared with respect to age, sex, vaccination status, and region. Rates were calculated by using 2001 population estimates from the Office for National Statistics. The incidence rate for cases confirmed positive by oral fluid was adjusted for the proportion of reported cases tested in each region and compared with the rate from laboratory reports alone. All analyses were conducted by using Stata/SE version 9.2 (StataCorp LP, College Station, TX, USA). Proportions were compared by using the χ^2^ test.

## Results

### Accuracy of Clinical Case Definition

During January 1995–July 2003, of 29,825 reported case-patients, oral fluid from 17,042 (57%) was tested for IgM; complete clinical information was obtained for 12,220 (72%) patients who submitted oral fluid samples. The overall sensitivity of the clinical case definition of maculopapular rash and fever and lymphadenopathy was 51% (95% confidence interval 48.9%–54.0%) and the specificity was 55% (95% confidence interval 53.7%–55.6%). The sensitivity and specificity of this case definition did not show significant variation by age, sex, and year of reporting (Table 1). However, the positive predictive value was significantly higher for persons >15 years of age (71% for persons 15–24 years of age compared with 1% for persons 5–9 years of age), for men (21% compared with 5% for women), and during the 1995–1998 epidemic period (20% compared with 1% in January 1999–July 2003).

**Table 1:** Accuracy of World Health Organization–modified clinical case definition for rubella, England and Wales, 1999–2008*


Characteristic	Sensitivity			Specificity			Positive predictive value	
	No. positive/ no. tested	% (95% CI)		No. positive/ no. tested	% (95% CI)		No. positive/ no. tested	% (95% CI)
Age, y								
<1	22/37	59.5 (43.6–75.3)		1,467/2,804	52.3 (50.5–54.2)		22/1,359	1.6 (1.0–2.3)
1–4	34/75	45.3 (34.1–56.6)		2,303/4,522	50.9 (49.5–52.4)		34/2,253	1.5 (1.0–2.0)
5–9	7/11	63.6 (35.2–92.1)		1,006/1,640	61.3 (59.0–63.7)		7/641	1.1 (0.3–1.9)
10–14	10/32	31.3 (15.2–47.3)		298/426	70.0 (65.6–74.3)		10/138	7.2 (2.9–11.6)
15–24	433/871	49.7 (46.4–53.0)		281/462	60.8 (56.4–65.3)		433/614	70.5 (66.9–74.1)
>25	256/455	56.3 (51.7–60.8)		257/439	58.5 (53.9–63.2)		256/438	58.4 (53.8–63.1)
Sex								
M	649/1,278	50.8 (48.0–53.5)		2,877/5,326	54.0 (52.7–55.4)		649/3,098	20.9 (19.5–22.4)
F	112/202	55.4 (48.6–62.3)		2,744/4,954	55.3 (54.0–56.8)		112/2,322	4.8 (3.95–5.7)
Year of report								
1995 Jan–1998 Dec	743/1,435	51.8 (49.2–54.4)		3,630/6,677	54.4 (53.2–55.6)		743/3,790	19.6 (18.3–20.9)
1999 Jan–2003 Jul	18/46	39.1 (25.0–53.2)		1,983/3,616	54.8 (53.2–56.5)		18/1,651	1.1 (0.6–1.6)

*CI, confidence interval.

### Enhanced Surveillance, 1999–2008

During 1999–2008, a total of 13,952 clinically suspected rubella cases were reported, and the number of cases per year (1,000–2,000) remained stable (Figure). Oral fluid was tested for 11,709 (84%) case-patients; 143 (1.0%) positive results were confirmed, and 11,566 (99%) of the results were discarded (Table 2) This finding is equivalent to an annual investigation and discard rate of 2,208 clinically suspected rubella cases per 100,000 population. Over the 10-year period, the proportion of confirmed cases for which oral fluid was tested increased from 39% (49/127) in 1999 to 49% (16/33) in 2008. The annual number of cases confirmed positive by oral fluid test remained <30 (Figure). The proportion of cases with a confirmatory test result has decreased since 1999 and remained <2%, except for a temporary increase in 2007 to 2.8%. During the 10-year period, 263 additional cases were confirmed by serum testing, resulting in 406 confirmed cases compared with 13,952 clinically diagnosed cases. Because laboratories in England and Wales report only cases that were confirmed positive, the number of additional cases tested and results discarded after serum testing alone is not known.

**Figure:**
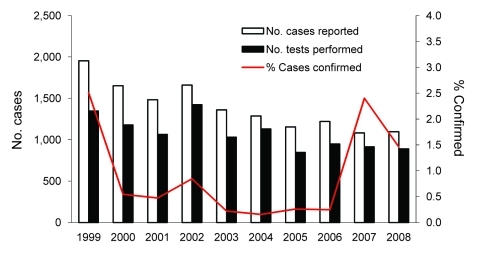
Number of rubella cases reported, number of oral (crevicular) fluid tests performed, and proportion of reported cases confirmed by oral fluid testing, England and Wales, 1999–2008.

**Table 2:** Reported and confirmed rubella cases in enhanced surveillance program, England and Wales, 1999–2008*

Age, y	Total no. reports					No. cases confirmed by oral (crevicular) fluid testing					No. additional cases confirmed by serum testing			
	M	F	UNK	Total no. (%)		M	F	UNK	Total no. (%)		M	F	UNK	Total no. (%)
<1	1,823	1,674	23	3,520 (25.0)		10	10	1	21 (15.0)		4	2	1	7 (2.7)
1–4	3,406	2,882	37	6,325 (45.0)		14	9	1	24 (17.0)		19	10	1	30 (11.0)
5–9	1,083	1,005	14	2,102 (15.0)		2	3	0	5 (3.5)		2	2	0	4 (1.5)
10–14	339	298	4	641 (4.6)		3	1	0	4 (0.3)		7	3	1	11 (4.2)
>15	506	729	7	1,242 (8.9)		63	26	0	89 (62.0)		93	109	7	209 (79.0)
UNK	54	56	12	122		0	0	0	0		0	2	0	2
Total no. (%)	7,211 (52.0)	6,644 (48.0)	97	13,952		92 (64.0)	49 (34.0)	2	143		125 (48.0)	128 (48.0)	10	263
*UNK, unknown.														

The age and sex distribution of case-patients reported from each surveillance source differed markedly (Table 2). The proportion of reported cases for which oral fluid was tested was higher for children <15 years of age (10,763/12,588 [86%]) than for persons >15 years (717/1,242 [58%]). However, cases in children <15 years of age were significantly less likely to be confirmed positive by oral fluid testing than were cases in persons >15 years of age (54/10,763 [0.5%] vs. 89/717 [12%]; p = 0.0001). Adults represented the largest proportion of all serum-confirmed case-patients during the 10-year period. However, a higher proportion of cases for which oral fluid was tested were in children <15 years of age than cases confirmed by serum testing (54/89 [61%] vs. 52/209 [25%], respectively; p = 0.0001).

Reports of rubella showed an approximately equal sex distribution among persons <15 years of age but showed a slight preponderance of female patients among adults (Table 2). The proportion of reported cases for which oral fluid was tested did not differ by sex (6,084/7,211 [84%] male patients and 5,521/6,644 [83%] female patients). The proportion of cases confirmed positive by oral fluid testing was similar for boys and girls <15 years of age but significantly higher for men than for women (63/265 [24%] vs. 26/442 [5.9%]; p = 0.0001). Cases confirmed by serum testing showed an equal sex distribution, whereas cases confirmed by oral fluid testing showed a predominance of male patients (125/253 [49%] and 92/141 [65%] male patients among serum-confirmed and oral fluid–confirmed cases, respectively; p = 0.002).

Data sources also differed with respect to vaccination status. The proportion of reported cases that could be confirmed as rubella was almost 7× higher for unvaccinated than for vaccinated persons (128/6,543 [2.0%] and 15/5,122 [0.3%], respectively; p<0.0002). However, the absolute difference varied with age. Although a small but significant difference occurred between unvaccinated and vaccinated children <5 years of age (40/5,515 [0.7%] and 5/3,007 [0.2%], respectively; p = 0.0007), the difference was much larger in persons 15–24 years of age (46/150 [31%] and 2/171 [1.2%], respectively; p<0.0001).

Regional distribution varied by data source (Table 3). The annual incidence based on reported cases was highest in the North East region and lowest in Wales, whereas the annual incidence based on laboratory reports of serum confirmation was highest in London. Incidence based on oral fluid test results also differed, even after adjustment for the proportion of reported cases tested. The estimated overall incidence of confirmed rubella increased by 54% (from 0.50 cases/1 million population to 0.77 cases/1 million population) when data for oral fluid testing were included. Oral fluid data also changed the ranking of regions; the Eastern region overtook London in reporting the highest overall incidence. Although the West Midlands and Yorkshire and Humberside regions reported the lowest incidence on the basis of serum testing alone, after oral fluid testing was included, the East Midlands region reported the lowest overall incidence.

**Table 3:** Regional variation in rubella reports by oral (crevicular) fluid testing and confirmed cases from oral fluid and serum, England and Wales, 1999–2008

Region	No. reported cases	Incidence of reported cases*	No. oral fluid tests (% total reports)	No. cases confirmed by oral fluid testing	No. cases confirmed by serum testing	Annual incidence of confirmed cases*		
						Confirmed by oral fluid testing†	Confirmed by serum testing	Total
East Midlands	1,336	319	1,065 (80)	3	9	0.09	0.21	0.29
Eastern	1,212	224	1,232 (102)‡	44	32	0.80	0.59	1.41
London	1,653	226	1,452 (88)	32	67	0.67	0.92	1.35
North East	840	331	574 (68)	0	12	0.00	0.47	0.47
North West	1,662	245	1,505 (91)	12	13	0.25	0.19	0.37
South East	2,363	295	2,350 (99)	18	48	0.34	0.60	0.82
South West	1,109	224	857 (77)	13	40	0.31	0.81	1.07
West Midlands	1,235	234	821 (66)	12	10	0.33	0.19	0.42
Wales	639	220	518 (81)	1	10	0.02	0.34	0.38
Yorkshire and Humberside	1,903	382	1,260 (66)	7	22	0.20	0.44	0.58
Not specified			75	1	0			
Total	13,952	266	11,709 (84)	143	263	0.31	0.50	0.77

*Incidence per 1 million population. Annual incidence calculated by using 2001 census population figures. †Adjusted for proportion of cases tested. ‡Several oral fluid tests were conducted for cases that were not formally reported during university outbreaks in 3 regions in 1999, including the South West, Eastern, and North West regions (*17*).

## Discussion

Before vaccination was introduced, epidemics of rubella occurred regularly and caused mild rash illness, predominantly in children. In 1970, introduction of a selective vaccination policy in the United Kingdom aimed to reduce the risk for infection in early pregnancy and the risk for fetal death and CRS. Despite the success of the selective policy, MMR was adopted into the routine childhood vaccination schedule to eliminate circulating rubella and to further reduce the risk for CRS.

Since 1988, a clinical diagnosis of rubella has been reportable by registered medical practitioners in England and Wales under the statutory Notification of Infectious Diseases; there is no case definition. When an infection is commonly occurring in an area, the positive predictive value of a clinical diagnosis may be sufficient for accurate surveillance (*24*). However, because rubella has become less common, an increasing proportion of reported cases are likely to be caused by other infections that have similar clinical manifestations. The rash of rubella may be temporary and can resemble the rash caused by other viruses. For example, infection with parvovirus B19, human herpesvirus 6 (roseola infantum), and human herpesvirus 7 all involve rash and fever and may be misdiagnosed as rubella (*25**,**26*).

We have confirmed the low sensitivity and specificity of the clinical case definition and that this definition is not affected by age, sex, and period of reporting. Despite some missing clinical information and the absence of information about arthralgia, this finding suggests that the WHO clinical case definition is not sufficiently accurate for surveillance in the postvaccine era. This finding also emphasizes the need to have laboratory confirmation of all clinically diagnosed cases to accurately monitor rubella elimination (*27*).

Over the 10-year period of elimination, only ≈1 of 100 persons reported with clinically diagnosed rubella and who underwent oral fluid testing had confirmed cases. In addition, reported cases differed from laboratory-confirmed cases with respect to patient age, sex, vaccination status, and geographic distribution. We have therefore shown that surveillance based only on clinical reports would substantially overestimate the true incidence of rubella, particularly in children, and therefore give a misleading epidemiologic picture. Furthermore, we have shown that testing of oral fluid is acceptable in the United Kingdom and can be used to augment routine serologic diagnosis. Approximately one third of confirmed rubella cases were diagnosed by testing of oral fluid, which improved ascertainment of confirmed infections in children and men. In addition, by using PCR, we obtained genotype information on 12 samples that would have otherwise not been available.

Currently, there is only 1 commercial assay for testing rubella IgM in oral fluid, and this assay does not have an In Vitro Diagnostics license, thus limiting its use in some countries. However, in many regions, WHO is evaluating this assay as a tool for surveillance of infection (*28*; K. Brown, pers. comm.) Although the UK system relies upon a well-organized postal service, alternative approaches for delivering and receiving specimens directly from the patient’s home or family practice may be required in other countries considering the use of oral fluid.

In addition to antibody testing, oral fluid can be used for rubella RNA detection in samples obtained early during infection (*13*) and for genotyping and molecular epidemiologic studies (*14**,**15*). Tests for detecting rubella-specific IgM and RNA in oral fluid samples are also suitable for confirming a diagnosis of CRS (*11*). The same system is also being used to monitor measles and mumps incidence and to inform MMR vaccine policy in England and Wales (*18**,**19**,**29**,**30*).

Oral fluid testing can also be used to evaluate the completeness of rubella surveillance. Since 1999, the number of reports of rubella and oral fluid tests performed annually has remained constant, and the proportion of cases tested has remained high. When combined with a low rate of confirmation, including cases diagnosed as clinical rubella, in a country with free universal access to high-quality primary-care physicians, this finding suggests that surveillance of confirmed rubella is nearly complete in England and Wales. Despite this suggestion, annual incidence of confirmed rubella remains <1 per 1 million population, which is the goal for elimination (*4*).

In recently published surveillance guidelines, WHO has recommended IgM detection, which can be performed with serum and oral fluid (*31*). These guidelines also describe performance indicators to assess the quality of national surveillance systems in the elimination phase. These indicators include a laboratory investigation rate (proportion of clinically suspected cases with adequate specimens for IgM testing) >80% and a detection rate for the number of clinically suspected rubella cases investigated and discarded by laboratory testing >2/100,000 population/year. Data from the enhanced surveillance program show that for ≈84% of reported cases, oral fluid was tested, and 2,208 clinically suspected rubella cases per 100,000 population were investigated and discarded. This high discard rate would be feasible only with noninvasive testing and contributes to the high quality of the UK enhanced surveillance program. Information about the low rate of rubella supplements surveillance that confirms that CRS incidence in the United Kingdom was 0.14/100,000 live-born infants in 2007 (*32*), which was far below the WHO elimination goal of 1/100,000 live-born infants (*4*).

We confirmed that a clinical case definition alone is not sufficiently specific for surveillance of rubella in the elimination era and that laboratory confirmation by testing serum samples is biased and incomplete. Since 1999, a substantial proportion of confirmed cases of rubella identified through the enhanced surveillance scheme have occurred in unvaccinated men. With the availability of oral fluid testing, a high number and high proportion of suspected cases have been tested. However, numbers of confirmed rubella cases in children remain low, which is consistent with high levels of vaccine coverage and low levels of susceptibility in this younger age group (*33*). The enhanced oral fluid surveillance system has proven valuable for accurately assessing progress toward achieving the WHO goal of eliminating circulating rubella and CRS from the population of England and Wales.
